# Fabrication and Characterization of Finite-Size DNA 2D Ring and 3D Buckyball Structures

**DOI:** 10.3390/ijms19071895

**Published:** 2018-06-27

**Authors:** Soojin Jo, Seungjae Kim, Byung Ho Lee, Anshula Tandon, Byunghoon Kim, Sung Ha Park, Moon Ki Kim

**Affiliations:** 1 School of Mechanical Engineering, Sungkyunkwan University, Suwon 16419, Korea; jsj89@skku.edu (S.J.); byunghooo@gmail.com (B.H.L.); 2 Department of Physics, Sungkyunkwan University, Suwon 16419, Korea; seungjae@skku.edu (S.K.); werft2@skku.edu (B.K.); 3 Sungkyunkwan Advanced Institute of Nanotechnology (SAINT), Sungkyunkwan University, Suwon 16419, Korea; anshula.tandon@gmail.com

**Keywords:** finite-size, DNA structure, self-assembly, elastic network model, normal mode analysis

## Abstract

In order to incorporate functionalization into synthesized DNA nanostructures, enhance their production yield, and utilize them in various applications, it is necessary to study their physical stabilities and dynamic characteristics. Although simulation-based analysis used for DNA nanostructures provides important clues to explain their self-assembly mechanism, structural function, and intrinsic dynamic characteristics, few studies have focused on the simulation of DNA supramolecular structures due to the structural complexity and high computational cost. Here, we demonstrated the feasibility of using normal mode analysis for relatively complex DNA structures with larger molecular weights, i.e., finite-size DNA 2D rings and 3D buckyball structures. The normal mode analysis was carried out using the mass-weighted chemical elastic network model (MWCENM) and the symmetry-constrained elastic network model (SCENM), both of which are precise and efficient modeling methodologies. MWCENM considers both the weight of the nucleotides and the chemical bonds between atoms, and SCENM can obtain mode shapes of a whole structure by using only a repeated unit and its connectivity with neighboring units. Our results show the intrinsic vibrational features of DNA ring structures, which experience inner/outer circle and bridge motions, as well as DNA buckyball structures having overall breathing and local breathing motions. These could be used as the fundamental basis for designing and constructing more complicated DNA nanostructures.

## 1. Introduction

DNA has emerged as a fascinating molecule for self-assembly in structural DNA nanotechnology due to its inherent molecular recognition, base-sequence programmability, well-structured conformation, and predictable nanoscale dimensions [1,2,3]. The essence of this burgeoning field is the construction of a wide variety of novel structures via self-assembly of DNA building blocks on the nanometer scale with high precision. Self-assembly of these nucleic acid building blocks can be used to design and construct various dimensional nanostructures. To date, there have been many experimental attempts to construct various DNA nanostructures including one-dimensional (1D) tubes [4], 2D lattices [5,6,7,8], 2 and 3D molecular canvases [9,10,11], as well as finite-size 2D rings [12,13] and 3D polyhedra [14,15,16,17].

Simulation-based studies are useful for obtaining clues related to the geometric stabilities, specific functionalities, and dynamic characteristics of DNA nanostructures. Normal mode analysis (NMA) has been widely used to analyze intrinsic modes of biomolecules due to its wide adaptability and relatively low cost. Collective motions of biomolecules have a significant influence on their structural characteristics [18,19,20,21]. There have been considerable efforts to analyze vibrational characteristics based on NMA for various DNA/RNA native structures [22,23,24,25,26]. Although dynamic analysis for some fabricated nanostructures has also been performed by NMA [20,21,27,28,29,30,31], finite-size nanostructures that have more complex geometries, as well as larger molecular weights, have rarely been studied.

In this study, we introduced two representative finite-size DNA nanostructures, i.e., 2D rings and 3D buckyballs, and performed computational analysis on them using NMA based on a mass-weighted chemical elastic network model (MWCENM) and a symmetry-constrained elastic network model (SCENM) [21,32]. Intrinsic vibrations of the DNA 2D ring and 3D buckyball structures can be determined precisely and efficiently because MWCENM considers both molecular weights and chemical bonds, and SCENM uses the information of a repeated unit and connectivity between adjacent units.

## 2. Results

### 2.1. DNA 2D Ring and 3D Buckyball Synthesis Results

**Figure 1:**
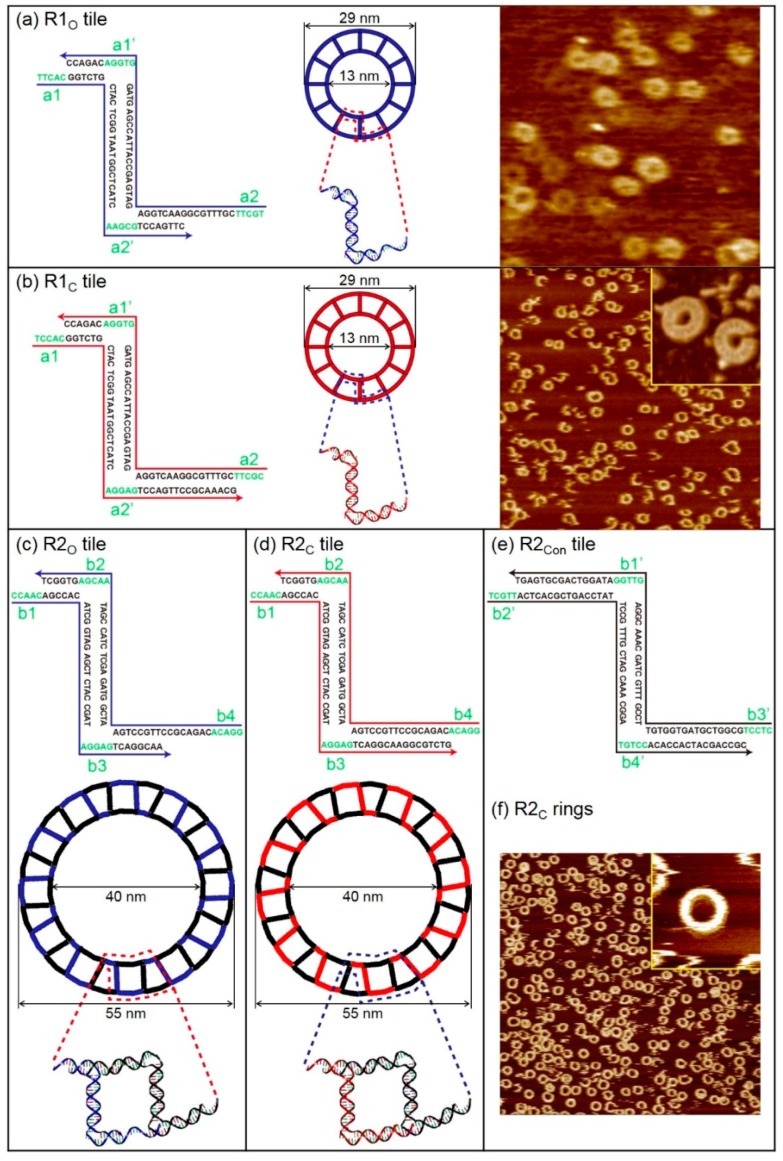
Schematics of finite-size DNA 2D ring structures with their unit tile sequences and their representative atomic force microscope (AFM) images. (**a**) DNA base sequence of the open-formed unit building block (referred to as an R1_O_ tile), schematic diagram, and corresponding AFM images of DNA 2D ring structures composed of R1_O_ tiles (referred to as an R1_O_ ring) indicated in blue. The complementary sticky end pairs are shown as a# and a#’. The outer and inner diameters of DNA rings are 29 nm and 13 nm, respectively. Here, the scan size of the image is 500 × 500 nm^2^. (**b**) DNA base sequence of the closed-formed unit building block (referred to as an R1_C_ tile), schematic diagram, and corresponding AFM images of DNA 2D ring structures composed of R1_C_ tiles (referred to as an R1_C_ ring) indicated in red. Scan sizes of the image and the magnified image in the inset are 1 × 1 μm^2^ and 80 × 80 nm^2^, respectively. (**c**–**e**) DNA base sequences of open-formed (closed-formed) and connection unit building blocks (referred to as R2_O_ (R2_C_) and R2_Con_ tiles, respectively) and schematic diagram of the DNA 2D ring structure composed of R2_O_ (R2_C_) and R2_Con_ tiles (referred to as an R2_O_ (R2_C_) ring) indicated in blue (red) and black, respectively. The complementary sticky end pairs are shown as b# and b#’. The outer and inner diameters of the DNA rings are 55 nm and 40 nm, respectively. (**f**) Representative AFM image of R2_C_ rings. Scan sizes of the image and the magnified image in the inset are 2 × 2 μm^2^ and 100 × 100 nm^2^, respectively.

Finite-size DNA 2D ring structures, i.e., open- and closed-formed configurations, were fabricated as shown in Figure 1. An open-formed ring (referred to as the R1_O_ ring) is made of 12 units of R1_O_ tiles. Each tile contains an eight-nucleotide long single-stranded domain that provides the flexibility of a duplex. In contrast, a closed-formed ring (referred to as the R1_C_ ring) does not have a single-stranded domain. Each R1_O_ and R1_C_ tile has two sticky end pairs (known as the complementary sticky ends shown as a# and a#’ in Figure 1a,b) for hybridization. Both rings have outer and inner diameters of 29 nm and 13 nm, respectively. DNA base sequences of R1_O_ (indicated in blue) and R1_C_ (red) rings and the corresponding atomic force microscope (AFM) images (dimensions are in good agreement with the design) are shown in Figure 1a,b. In addition, R2 rings (R2_O_ or R2_C_ rings) containing connection tiles (referred to as R2_Con_ tiles), which make a ring relatively larger than R1 rings, were constructed in order to demonstrate the feasibility of designing various DNA ring diameters and study size-dependent vibrational characteristics. A representative AFM image of R2_C_ rings, which agreed with the designed dimensions (the outer and inner diameters of DNA rings are 55 nm and 40 nm, respectively), is shown in Figure 1c–f.

**Figure 2:**
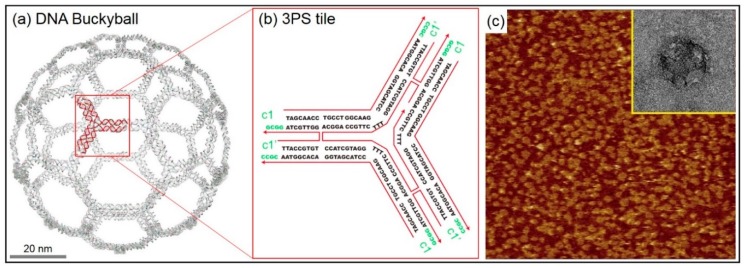
Finite-size DNA 3D buckyball structure with its unit tile sequence, representative atomic force microscope (AFM) and transmission electron microscope (TEM) images. (**a**) Schematic diagram of a DNA 3D buckyball structure composed of three-point-star (3PS) unit tiles. (**b**) DNA base sequence of a 3PS unit tile. The complementary sticky end pairs are shown as c1 and c1’. (**c**) AFM and TEM images of the DNA 3D buckyball structure. Scan sizes of the AFM and the TEM images in the inset are 3 × 3 μm^2^ and 100 × 100 nm^2^, respectively.

Finite-size DNA 3D buckyball structures that consisted of 60 three-point-star (3PS) building blocks (consisting of seven individual strands with threefold rotational symmetry) were constructed as shown in Figure 2. The buckyball represents a cage-like structure composed of 20 hexagons and 12 pentagons containing 60 vertexes, 90 edges, and 32 faces. In a 3PS tile, a long central strand with three single-stranded T-loops (provided spatial flexibility) and single-stranded overhangs (that serve as sticky ends) allowed the formation of a 3D geometry, i.e., a DNA buckyball. AFM and transmission electron microscope (TEM) images of the DNA 3D buckyball structure are shown in Figure 2c. Although the buckyball structures shown in the AFM image were not guaranteed to possess 60 3PS tiles, the diameter of the structure (~80 nm) roughly agreed with the designed scheme.

### 2.2. Normal Mode Analysis Results of DNA 2D Ring and 3D Buckyball Structures

Three-dimensional computer models were needed to analyze vibrational characteristics of DNA 2D ring and 3D buckyball structures. For 2D rings, we can construct their structures by using only their unit tile coordinates with 12 rotation matrices because they have 12-fold ring structures. After modeling different unit tiles of R1_O_, R1_C_, R2_O_, and R2_C_ rings on the xy-plane based on their base sequences, their entire ring structures were constructed by rotating every 30° about the *z*-axis. Likewise, we can construct the 3D buckyball structure by using its unit tile coordinates with 60 rotation matrices because it is an icosahedron symmetry structure with 60 repeated 3PS unit tiles. First, the 3PS unit tile was generated on the xy-plane based on its base sequence. The whole buckyball structure was constructed using the 3D coordinates of the unit tile and 60 rotation matrices referenced from the HK97 virus structure, which also consisted of 60 repeated units [33]. In order to verify appropriate assignment between adjacent unit tiles in the constructed buckyball, the number of hydrogen bonds at their sticky ends was evaluated. After modeling finite-size 2D rings and 3D buckyball, we confirmed their structural reliabilities by calculating both the hydrogen bond length between complementary base pairs and distances between two adjacent atoms. Appropriate binding between unit tiles was verified in all 3D computer models and no steric clashing was found.

**Figure 3:**
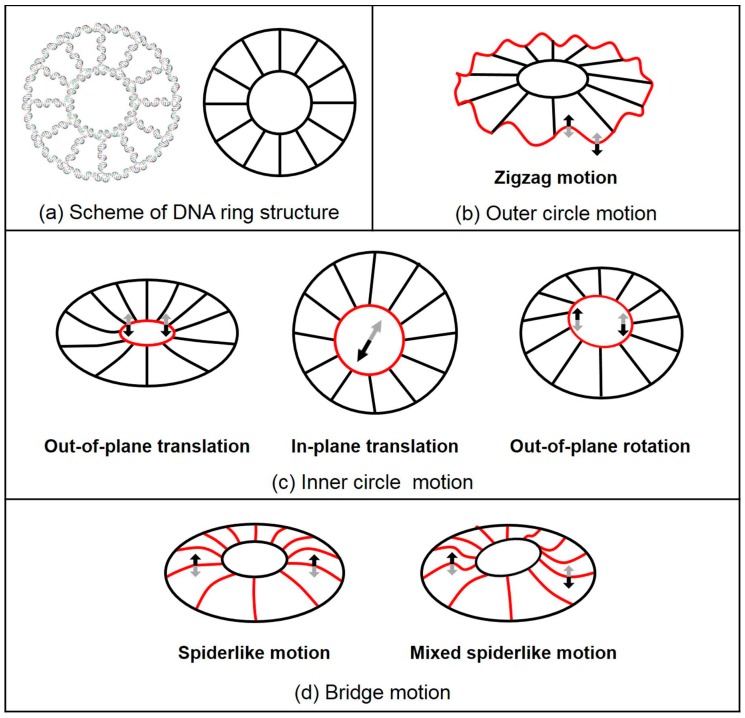
Schematics of the DNA 2D ring structure and its major vibration modes. These modes are classified into three different motions, including outer circle motion, inner circle motion, and bridge motion. (**a**) Scheme of the DNA ring structure. (**b**) Zigzag motion, outer circle motion, is sinusoidal and the shape of outer circle is red. The 8th–10th mode at R1 rings and the 4th–10th mode at R2 rings are shown. (**c**) Inner circle motions consist of out-of-plane translation, in-plane translation, and out-of-plane rotation of inner circle (indicated in red). Out-of-plane and in-plane translations are the inner circle of the DNA ring structure moving in vertical and lateral directions, respectively. Out-of-plane rotation is the seesaw motion of the inner circle. For all ring structures, out-of-plane translation and out-of-plane rotation are dominant motions, as shown in the 1st mode and both the 2nd and 3rd modes, respectively. In-plane translation can be observed only at R1 rings over the 7th mode. (**d**) Bridge motion is composed of spiderlike and mixed spiderlike motions. Spiderlike motion is the bridge bending motion in the upper direction and is observed in all ring structures. It is present in the 4th mode of R1 rings. Mixed spiderlike motion is also shown in both the 5th and the 6th modes of the R1 rings.

Both MWCENM and SCENM were then applied to these 3D computer models to obtain their intrinsic vibration modes. Figure 3 shows the major vibrational modes of 2D rings. Their dominant modes are classified into three different motions, including the outer circle motion (i.e., zigzag motion), inner circle motion (i.e., out-of-plane translation, in-plane translation, and out-of-plane rotation of the inner circle), and bridge motion (i.e., spiderlike and mixed spiderlike motions). The first three lowest modes show inner circle motions for all 2D rings, such as out-of-plane translation of the inner circle at the 1st mode and out-of-plane rotation of the inner circle at both the 2nd and the 3rd modes. At the 4th mode, spiderlike motion and zigzag motion were present at the R1 rings (R1_O_ and R1_C_ rings) and R2 rings (R2_O_ and R2_C_ rings), respectively. Bridge motions appeared more frequently in R1 rings, but R2 rings had no bridge motion below the 8th lowest mode. Also, the outer circle motions appeared more frequently in R2 rings compared to R1 rings. In-plane translation of the inner circle was shown only at R1 rings higher than the 7th mode. Over the 9th mode, the mixed outer circle and bridge motions or outer circle and inner circle motions were observed in all 2D rings. Therefore, DNA 2D rings showed six different major vibrational modes. Overall, their vibrational characteristics depended on their size, and there was no significant difference between the open-formed and closed-formed configurations.

**Figure 4:**
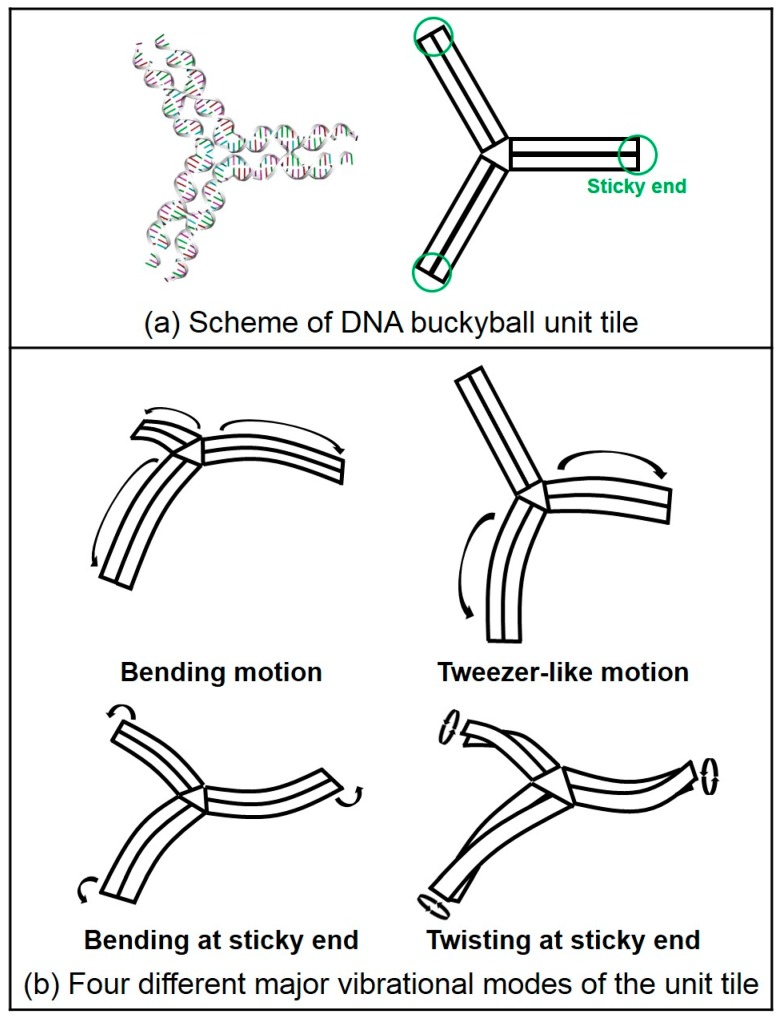
Schematics of three-point-star (3PS) unit tile and its major vibration modes. (**a**) Scheme of 3PS unit tile. DNA duplex is represented as a rectangle, and its three sticky ends are marked with green circles. (**b**) The unit tile shows four different major vibrational modes such as bending motion, tweezer-like motion, and bending or twisting at sticky ends. Within the 10th lowest mode, the bending motion is the 1st mode and both the 3rd and the 8th modes are tweezer-like motions. Other modes show bending, twisting or mixed bending and twisting at sticky ends.

**Figure 5:**
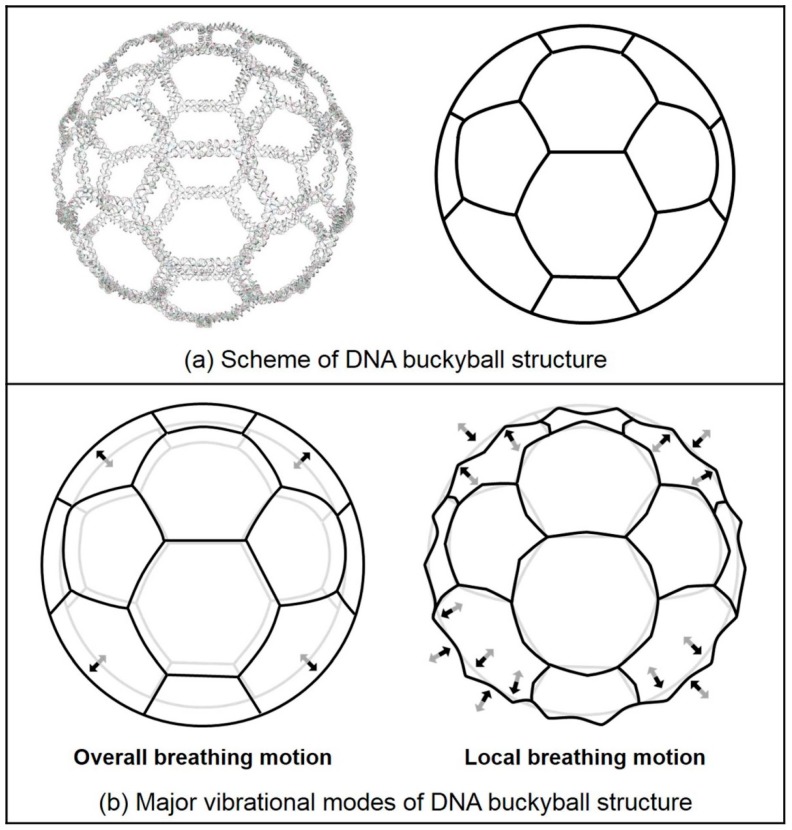
Schematics of the DNA buckyball structure and its major vibration modes. (**a**) The scheme of the DNA buckyball is represented as soccer ball. (**b**) Major vibrational modes of the DNA buckyball are overall breathing motion and local breathing motion. Overall breathing motion is the swelling of the entire buckyball structure, and local breathing motion forms a spiky shape. Its conformational change from initial to final structures is depicted in gray and black, respectively.

In addition, we calculated normal modes for both the buckyball and its 3PS unit tile structures. Vibrational mode shapes of the unit tile structure can be easily obtained by using MWCENM-based NMA. In the case of the 3D buckyball structure, however, we additionally adopted the SCENM method based on its icosahedral symmetry in order to reduce the computational cost in NMA due to its large structural size (i.e., the number of coarse-grained atoms is 102,960). Finally, we successfully obtained mode shapes of the 3D buckyball structure by using only the unit tile coordinates along with its information related to connectivity with neighboring tiles. These results are shown in Figure 4 and Figure 5, respectively. Figure 4 shows the major vibrational modes of the 3PS unit tile. It primarily shows bending motion, tweezer-like motion, and twisting or bending at the sticky ends. The bending motion is on the 1st lowest mode, and tweezer-like motion appeared at the 3rd and the 8th modes. Other modes below the 10th lowest mode are bending, twisting, and mixed motion of bending and twisting at sticky ends. Based on these dominant modes of the unit tile, we found that 3PS unit tiles can be assembled as spherical buckyball structures. Figure 5 shows the dominant vibrational modes of the whole buckyball structure. These major modes are overall breathing and local breathing motions. The overall breathing motion appeared at the 1st and the 2nd modes, and the local breathing motion was observed from the 4th–7th modes. Over the 8th mode, the mixed local breathing and other motions were observed. Uncommon torsional motion appeared at the 3rd mode. In this mode, the motion of the unit tile is composed of two stationary sticky ends and one twisting sticky end. The first 10 lowest modes for all finite-size DNA rings and buckyball structures are summarized in Table 1.

## 3. Discussion

In summary, we designed, fabricated, and characterized finite-size DNA 2D ring and 3D buckyball structures. DNA 2D ring and 3D buckyball structures were successfully fabricated, and their physical geometries were verified through AFM and TEM. In addition, 3D computer models were constructed for these structures, which were then used to characterize their vibrational features based on both MWCENM- and SCENM-based NMA. When establishing 3D computer models, their structural reliability was confirmed by evaluating the number of hydrogen bonds and distances between adjacent atoms. The 2D ring results of NMA showed that major vibration modes were categorized into three different motions, including inner circle motion, outer circle motion, and bridge motion. Although size-dependent vibrational properties were observed, there was no significant configuration difference between open- and closed-formed unit tiles. Both the unit tile and whole structures were analyzed for a 3D buckyball. Dominant vibration motions of the unit tile include bending, tweezer-like motion, and twisting or bending at the sticky ends. These modes seem to cooperate with each other to assemble spherical buckyball structures from unit tiles. Overall breathing and local breathing motions were mainly observed for the whole structure. In addition, the proposed NMA methodology is expected to play an important role in the construction of a structural and functional data library of various DNA nanostructures, as well as other macromolecules. This data library will lead to fundamental advances in simulation-based design, fabrication, and functionalization of DNA nanostructures by helping to understand their assembly mechanism and dynamics.

## 4. Materials and Methods

### 4.1. Synthesis Method

High-performance liquid chromatography purified synthetic oligonucleotides were purchased from BIONEER (Daejeon, Korea). DNA samples, i.e., DNA 2D ring and 3D buckyball structures, were formed by mixing a stoichiometric quantity of each strand in a buffer, 1× TAE/Mg^2+^ (40 mM Tris base, 20 mM acetic acid, 1 mM EDTA (pH 8.0), and 12.5 mM magnesium acetate). Final concentrations of 100 nM (for DNA 2D rings) and 500 nM (for 3D buckyball structures) were achieved. For annealing, the DNA strands were added to a test-tube with a total sample volume of 100 µL and were then placed in a Styrofoam box with 2 L of boiled water to cool slowly from 95 °C to 25 °C over a period of at least 24 h to facilitate hybridization.

### 4.2. AFM and TEM Measurements

For AFM imaging, the DNA sample was placed on a metal puck using instant glue. Then, 20 µL of 1× TAE/Mg^2+^ buffer was pipetted onto the sample substrate, and another 20 µL of 1× TAE/Mg^2+^ buffer was dispensed onto a silicon nitride AFM tip (Veeco Inc., San Jose, CA, USA). The AFM images were obtained by using a Multimode Nanoscope (Veeco Inc., USA) in liquid tapping mode. Transmission electron micrographs were obtained on a JEOL JEM-ARM300F for TEM imaging. Direct measurement of DNA 3D buckyball structures was carried out by pipetting 5 μL of an annealed DNA solution onto a carbon film-layered copper grid. Negative staining involved exposure of the sample on the grid to 10 μL of 2% uranyl acetate solution for a few minutes before imaging.

### 4.3. Mass-Weighted Chemical Elastic Network Model (MWCENM)

In an elastic network model (ENM), representative atoms are modeled as unit point masses, and connectivity between atoms is represented as a unit spring constant that varies depending on the cutoff distance, which is usually 12 Å in a *C_α_* coarse-grained protein model [34,35,36,37,38]. ENM methodology has been mainly used to analyze intrinsic mode shapes of proteins, but recently, it has also been used to analyze intrinsic vibrational features of DNA/RNA structures because of its advantages (i.e., low computational cost and large conformational change prediction) compared to the traditional full-atom molecular dynamics (MD) simulations [20,21,22,23,24,25,26,27,28,29,30]. In comparison with a traditional ENM, the MWCENM is a more precise modeling method because it considers both the inertia effect and the chemical bond information of the target system. The inertia effect is given by lumped masses to representative atoms of the target system, and chemical bonds are considered by assigning different spring constants, as shown in Table 2 [19,21]. Here, we assigned a lumped mass of the surrounding atoms to each representative atom. Each nucleotide is represented by six or seven atoms. Three atoms (P, C4, and C1) represent the sugar-phosphate backbone. In the pyrimidine (purine) base, three (four) atoms related to the connection with backbone or hydrogen bonds between complementary bases are selected as representative atoms [21]. A spring network of a coarse-graining model is constructed depending on the type of chemical bonds. Van der Waals interactions, hydrogen bonds, and covalent bonds are determined based on a cut-off distance of 8 Å, complementary base information, and DNA linkages, respectively, and their spring constant ratios are 1:10:100. Therefore, MWCENM enables us to calculate both the frequency values and corresponding mode shapes of the target system.

### 4.4. Symmetry-Constrained Elastic Network Model (SCENM)

If the target system has a symmetric structure composed of repeated subunit structures, we can use symmetry-constrained ENM (SCENM) to analyze full models effectively by using only a repeated subunit structure with connectivity information of neighboring subunits instead of handling all the structural information [32,39,40]. The advantage of SCENM is its ability to reflect the crystal packing effect of protein structures with reduced computational cost [41]. The computational cost is reduced as 1/*N* if the target structure consists of *N* copies of its subunit structure.

### 4.5. Normal Mode Analysis (NMA)

We performed MWCENM-based NMA to analyze vibrational characteristics of four different DNA 2D rings and the 3PS unit tile of a 3D buckyball. Suppose there is a system of *N* particles. Each particle is a representative atom chosen through sampling and a lumped mass that includes the weight of surrounding atoms. We constructed a spring network between particles using the chemical bond types (Table 2) and then constructed the equation of motion (EOM) for the system as follows. The position of the *i*th atom at time *t* $x_{i}\left(t\right)$ can be represented by the initial position $x_{i}\left(0\right)$ and a small displacement δ(t) so that $x_{i}\left(t\right)=x_{i}\left(0\right)+\delta \left(t\right)$, where $x_{i}\left(t\right)$ is a 3 × 1 vector. Then, the kinetic energy of the system can be defined as

(1) $$T=\frac{1}{2}\sum_{i=1}^{n}m_{i}{\left\|{\dot{x}}_{i}\left(t\right)\right\|}^{2},$$

where $m_{i}$ is the weight of a specific lumped mass. The kinetic energy can be rewritten in matrix form as

(2) $$T=\frac{1}{2}{\dot{\delta }}^{T}M\dot{\delta },$$

where *M* is a global mass matrix consisting of the lumped masses and δ is a 3*N* × 1 matrix. In addition, the potential energy can be written as

(3) $$V=\frac{1}{2}\sum_{i=1}^{n-1}\sum_{j=i+1}^{n}k_{i,j}{\left\{\|x_{i}\left(0\right)-x_{j}\left(0\right)\|-\|\delta_{i}\left(t\right)-\delta_{j}\left(t\right)\|\right\}}^{2},$$

where $k_{i,j}$ is the spring constant between the *i*th and *j*th atoms. By using a Taylor series approximation, we can rewrite Equation (3) as

(4) $$V=\frac{1}{2}\overset{n-1}{\underset{i=1}{\sum }}\overset{n}{\underset{j=i+1}{\sum }}k_{i,j}{\left(\delta_{i}\left(t\right)-\delta_{j}\left(t\right)\right)}^{T}\left(\frac{\left(x_{i}\left(0\right)-x_{j}\left(0\right)\right){\left(x_{i}\left(0\right)-x_{j}\left(0\right)\right)}^{T}}{\left\|x_{i}\left(0\right)-x_{j}\left(0\right)\right\|}\right)\left(\delta_{i}\left(t\right)-\delta_{j}\left(t\right)\right).$$

Then, we can derive the EOM using Lagrange’s equation.

(5) $$\frac{d}{dt}\left(\frac{\partial L}{\partial \dot{\delta_{i}}}\right)-\frac{\partial L}{\partial \delta_{i}}=0$$

Here, L=T−V. Therefore, the EOM for the system is

(6) $$M\ddot{\delta }+K\delta =0.$$

Next, we also performed SCENM-based NMA to analyze vibrational characteristics of the DNA 3D buckyball structure. Suppose that there is a system of *q* repeated subunits. We can then apply the symmetric boundary conditions to the system, such as intra-connectivity from a single subunit and the inter-connectivity from adjacent subunits. Because of the number of subunits *q*, we can rewrite $\vec{\delta }$ by dividing into *q* parts as

(7) $$\vec{\delta }={\left[{\vec{\Delta }}_{1}^{T},\cdots ,{\vec{\Delta }}_{q}^{T}\right]}^{T},$$

where $\Delta_{i}^{T}=\left[{\vec{\delta }}_{1}^{T},\cdots ,{\vec{\delta }}_{m}^{T}\right]$ is the *x*, *y*, *z* coordinates of a single subunit which has *m* atoms (*i* = 1, 2, …, *q*). If there are *q* rotation matrices for this structure, we can rewrite the coordinates of each subunit by using the first subunit and rotation matrices.

(8) $$\Delta_{i}=R_{i}\Delta_{1}$$

Here, $R_{i}=\left[\begin{array}{ccc} R^{\prime }{}_{1} & \cdots & 0\\ \vdots & \ddots & \vdots \\ 0 & \cdots & R^{\prime }{}_{m} \end{array}\right]$ is a rotation matrix between the first subunit and *i*th subunit and is a 3*m* by 3*m* matrix.

Then, we can construct the reduced stiffness matrix $K_{reduced}$ for a single subunit by including the connectivity information as

(9) $$K_{reduced}=K_{1,1}+K_{1,2}R_{2}+\cdots +K_{1,q}R_{q},$$

where $R_{2}$ and $R_{q}$ are rotation matrices, and $K_{1,1}$, $K_{1,2}$, and $K_{1,q}$ are the inter-connections between subunits. More details are described in Supplementary Material. Therefore, we can rewrite the EOM of the system to use $M_{reduced}$, which is an inertia matrix of a single subunit and $K_{reduced}$.

(10) $$M_{reduced}\ddot{\vec{\delta }}+K_{reduced}\vec{\delta }=0$$

Solving Equation (6) from MWCENM yields the vibrational motion and its frequency data. We can also obtain both vibrational motion and its frequency data of a single subunit by solving Equation (10) from SCENM. Vibrational motion of the whole structure can be recomposed to duplicate as a number of subunit copies. Otherwise, solving the procedure for the EOM is the same.

## Figures and Tables

**Table 1:** Vibrational mode shapes of finite-size DNA 2D ring and 3D buckyball structures.

Mode Number	Mode Shape				
	R1_o_ Ring	R1_c_ Ring	R2_o_ Ring	R2_c_ Ring	Buckyball
**Mode 1**	O-T ^a^ motion	O-T motion	O-T motion	O-T motion	O-B ^e^ motion
**Mode 2**	O-R ^b^ motion	O-R motion	O-R motion	O-R motion	O-B motion
**Mode 3**	O-R motion	O-R motion	O-R motion	O-R motion	Torsional motion
**Mode 4**	Spiderlike motion	Spiderlike motion	Zigzag motion	Zigzag motion	L-B ^f^ motion
**Mode 5**	M-S ^c^ motion	M-S motion	Zigzag motion	Zigzag motion	L-B motion
**Mode 6**	M-S motion	M-S motion	Zigzag motion	Zigzag motion	L-B motion
**Mode 7**	Spiderlike motion	I-T ^d^ motion	Zigzag motion	Zigzag motion	L-B motion
**Mode 8**	Mixed motion	I-T motion	Zigzag motion	Zigzag motion	Mixed motion
**Mode 9**	Mixed motion	Mixed motion	Mixed motion	Zigzag motion	Mixed motion
**Mode 10**	Mixed motion	Mixed motion	Zigzag motion	Mixed motion	Mixed motion

^a^ O-T: Out-of-plane translation; ^b^ O-R: Out-of-plane rotation; ^c^ M-S: Mixed spiderlike; ^d^ I-T: In-plane translation; ^e^ O-B: Overall breathing; ^f^ L-B: Local breathing.

**Table 2:** Various spring constants assigned to each type of chemical bond in MWCENM.

Chemical Bond	Spring Constant (N/m)
Van der Waals	7
Hydrogen bond	70
Ion bond	70
Disulfide bond	700
Covalent bond	700

## Supplementary Materials

Supplementary materials can be found at http://www.mdpi.com/1422-0067/19/7/1895/s1.
