# Supplementary material for: Fabrication and Characterization of Finite-Size DNA 2D Ring and 3D Buckyball Structures
Source: Int J Mol Sci. 2018 Jun 27;19(7):1895. doi: 10.3390/ijms19071895 (PMC6073519; doi:10.3390/ijms19071895)
Supplement: Supplementary file 1 [file ijms-19-01895-s001.pdf]

Supplementary Material

Vibrational Characteristics of DNA Nanostructures Obtained Through a Mass-weighted Chemical Elastic Network Model

Soojin Jo[1], Seungjae Kim[2], Byung Ho Lee[1], Anshula Tandon[3], Byunghoon Kim[2], Sung Ha Park[2,3,*], and Moon Ki Kim[1,3,*]

[1] School of Mechanical Engineering, Sungkyunkwan University, Suwon, 16419, Korea
[2] Department of Physics, Sungkyunkwan University, Suwon, 16419, Korea
[3] Sungkyunkwan Advanced Institute of Nanotechnology (SAINT), Sungkyunkwan University, Suwon, 16419, Korea

Symmetry-constrained elastic network model (SCENM)

How to construct the reduced connectivity matrix in SCENM. In this paper, in case of DNA buckyball structure, because of its icosahedral symmetric property, SCENM was additionally applied in order to effectively analyze intrinsic modes of buckyball structure by using only a three-point-star (3PS) tile with connectivity information of neighboring tiles instead of handling the whole buckyball structure. As shown in Figure S1, symmetric boundary condition including intraconnectivity from a single 3PS tile and interconnectivity from three neighboring tiles can be applied to construct the reduced connectivity matrix $K_{reduced}$ for a single 3PS tile such that

$$K_{reduced} = K_{1,1} + K_{1,2}R_2 + K_{1,3}R_3 + K_{1,4}R_4$$

Based on this methodology, the computational cost of normal mode analysis can be reduced by the factor of 60.

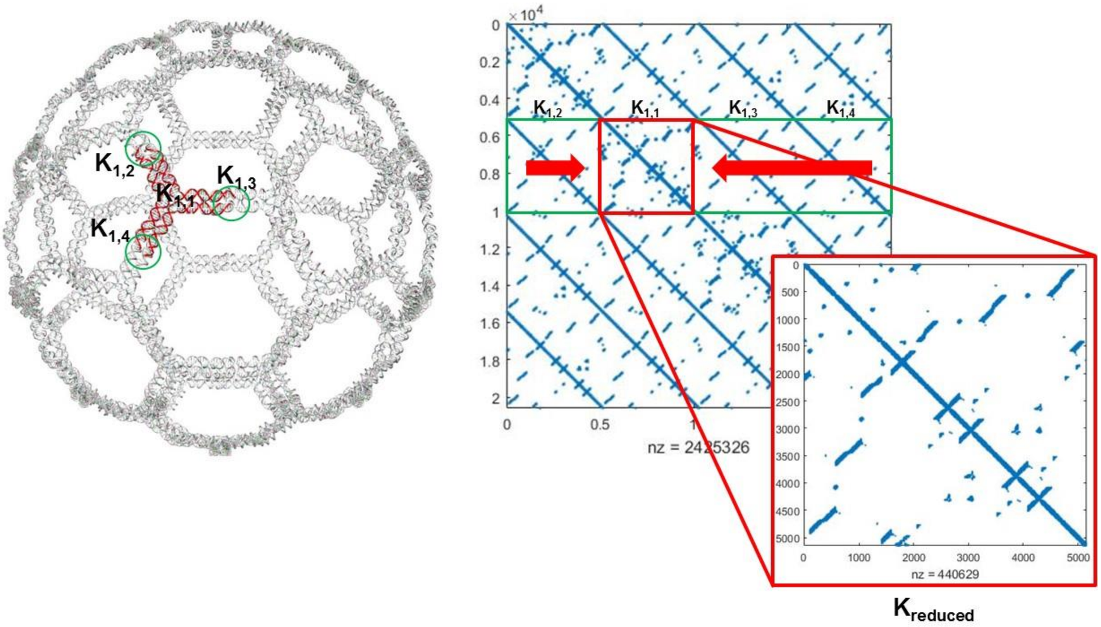

Figure S1. Connectivity information of the buckyball structure
